# Machine Learning Predicts Cerebral Vasospasm in Subarachnoid Hemorrhage Patients

**DOI:** 10.21203/rs.3.rs-3617246/v1

**Published:** 2024-02-05

**Authors:** David Zarrin, Abhinav Suri, Karen McCarthy, Bilwaj Gaonkar, Bayard Wilson, Geoffrey Colby, Robert Freundlich, Luke Macyszyn, Eilon Gabel

**Affiliations:** David Geffen School of Medicine; David Geffen School of Medicine; Department of Anesthesiology, Vanderbilt University Medical Center; UCLA Department of Neurosurgery; UCLA Department of Neurosurgery; UCLA Department of Neurosurgery; Vanderbilt University Medical Center; UCLA Department of Neurosurgery; UCLA

## Abstract

**Background:**

Cerebral vasospasm (CV) is a feared complication occurring in 20–40% of patients following subarachnoid hemorrhage (SAH) and is known to contribute to delayed cerebral ischemia. It is standard practice to admit SAH patients to intensive care for an extended period of vigilant, resource-intensive, clinical monitoring. We used machine learning to predict CV requiring verapamil (CVRV) in the largest and only multi-center study to date.

**Methods:**

SAH patients admitted to UCLA from 2013–2022 and a validation cohort from VUMC from 2018–2023 were included. For each patient, 172 unique intensive care unit (ICU) variables were extracted through the primary endpoint, namely first verapamil administration or ICU downgrade. At each institution, a light gradient boosting machine (LightGBM) was trained using five- fold cross validation to predict the primary endpoint at various timepoints during hospital admission. Receiver-operator curves (ROC) and precision-recall (PR) curves were generated.

**Results:**

A total of 1,750 patients were included from UCLA, 125 receiving verapamil. LightGBM achieved an area under the ROC (AUC) of 0.88 an average of over one week in advance, and successfully ruled out 8% of non-verapamil patients with zero false negatives. Minimum leukocyte count, maximum platelet count, and maximum intracranial pressure were the variables with highest predictive accuracy. Our models predicted “no CVRV” vs “CVRV within three days” vs “CVRV after three days” with AUCs=0.88, 0.83, and 0.88, respectively. For external validation at VUMC, 1,654 patients were included, 75 receiving verapamil. Predictive models at VUMC performed very similarly to those at UCLA, averaging 0.01 AUC points lower.

**Conclusions:**

We present an accurate (AUC=0.88) and early (>1 week prior) predictor of CVRV using machine learning over two large cohorts of subarachnoid hemorrhage patients at separate institutions. This represents a significant step towards optimized clinical management and improved resource allocation in the intensive care setting of subarachnoid hemorrhage patients.

## 1. Introduction

Cerebral vasospasm (CV) is a common angiographic finding following subarachnoid hemorrhage (SAH) and is widely reported as a primary contributor to delayed cerebral ischemia (DCI) and concomitant morbidity and mortality in this population ^1^. CV manifests with variable severity, being angiographically appreciable in up to 70% of all SAH patients and clinically symptomatic in 20–40% of SAH patients ^2,3^. A smaller subset of up to 20% of all CV patients suffer from severe CV which results in either death or severe neurologic deficit ^4^. The gold standard treatment for severe CV is angiographically guided injection of verapamil, a calcium channel blocker. Owing to the prevalence of severe CV after SAH, it is standard clinical practice to admit patients post-SAH to an ICU or neurocritical care unit (NCCU) for up to several weeks of vigilant, resource-intensive clinical monitoring ^5^.

The ability to stratify SAH by CV risk would allow for clinical monitoring commensurate to that risk, as opposed to indiscriminate monitoring of every patient with the same vigilance. Accurately predicting CVRV would allow for the redirection of clinical resources towards higher-risk cases while limiting ICU resources to those unlikely to have severe symptoms and negative outcomes. There have been many research attempts to predict or draw associations to CV, most using either human-generated radiographic scores, clinical scores, or molecular biomarkers as predictors^6–9^. Few studies have assessed the feasibility of predicting CV using ML ^10–17^, most achieving insufficient predictive accuracy for clinical rule-in/out of CV. Among the few studies achieving higher predictive accuracy, all were validated over small cohorts, were not externally validated, used a small portion of available clinical predictors, and relied on human-generated clinical scoring systems and subjectively interpreted radiographic findings, which are labor intensive and not available for all patients ^14,17,18^.

Our group was motivated to rigorously explore the utility of ML for vasospasm prediction in a large multi-center study. We tested the predictive accuracy of numerous machine learning networks trained on 172 unique ICU datapoints. We demonstrate the ability to predict CVRV with high accuracy using ML over the largest known cohort in the literature and over one week before the event occurs, and externally validate this model’s utility at a separate institution.

## 2. Methods

### 2.1 Data Extraction and Inclusion Criteria

This study qualified for UCLA IRB exception status (waiver of consent) because there was no direct contact with patients and all data in this study was de-identified. Data was extracted from the Perioperative Data Warehouse, which was developed by the UCLA Department of Anesthesiology and Perioperative Medicine and distributed to multiple centers across the United States ^19^. All patients with an ICD-10 code of I60 (nontraumatic SAH) or S06.6 (traumatic SAH) between 2013 and 2022 at UCLA were included.

De-identified clinical data spanning the entire hospital admission was extracted for each patient. This information included basic demographics, vitals, routinely collected clinical labs (complete blood count with differential, basic metabolic panel, arterial blood gas, Hemoglobin A1c (HgA1c), and cerebrospinal (CSF) analysis), intracranial pressure (ICP), respiratory variables (O2 flow, FiO2, EtCO2, airway grade, intubation attempts, nitric oxide), fluid status variables (volume of maintenance intravenous (IV) fluid, urine output, blood loss, blood administered), feeding (gastric feeding, emesis), and saturation (pulse oximetry, cerebral saturation)). Finally, the verapamil administration time was recorded if verapamil was administered. A complete list of clinical predictor variables is provided in **Supplemental Table I**.

### 2.2 Feature Extraction

For all patients, clinical data collected before ICU admission and after verapamil injection time or time-of-discharge from the ICU (depending on if verapamil was given) were excluded. ICP and mean arterial pressure (MAPs) time-series were encoded into a 20-dimensional feature vector containing values corresponding to the 5th, 10th, …, 95th, and 100th percentiles over the examined time period. Sera values such as complete blood count (CBC) results were encoded as a 5-dimensional feature vector representing the minimum, maximum, median, mean, and count of measurements. Additional “dependent variables” were derived from the originally extracted clinical variables, such as “total count of ICP measurements”.

### 2.3 Predictive Model Architecture, Training, and Cross Validation

We developed two models to aid in the prediction and interpretation of CVRV risk: a “prospective” predictive model and “retrospective” predictive model. The prospective model was developed to provide a clinically useful tool to assess CVRV risk in ICU patients. Prospective model predictions were performed using 4 hours, 1 day, 3 days, 5 days, 7 days, and 10 days of ICU data starting from the time of ICU admission. A “binary” prospective model was trained to predict between two outcomes, namely if the patient would need verapamil prior to ICU downgrade or not. A more advanced “trinary” prospective model was trained to predict amongst three outcomes, namely “will never get verapamil”, “will get verapamil within three days”, or “will get verapamil after three days” from the time of prediction.

Retrospective models were developed to explore the events preceding CVRV by analyzing variable importance scores in groups of patients at the same chronological stage prior to vasospasm. For the retrospective model, predictions were performed using all ICU data up until 4 hours, 1 day, 3 days, 5 days, 7 days, and 10 days before the prediction target: verapamil injection or ICU downgrade.

Each model was tested with two different predictor sets: an “institutional” and “conservative” (**Supplemental Table I**). The institutional predictor set contained all clinical variables, whereas the conservative predictor set contained only variables which are strictly measured in a highly standardized manner across medical institutions. Examples of such standardized variables include vital signs, routine lab values, ICP, etc.

The candidate network architectures tested were Logistic Regression, K Nearest Neighbors, Naïve Bayes, Decision Trees, Support Vector Machines, Gaussian Process Classifiers, Ridge Classifier, Random Forest Classifier, Quadratic Discriminant Analysis, Ada Boost Classifer, Gradient Boosting Classifier, Linear Extra Trees Classifier, Extreme Gradient Boosting, Light Gradient Boosting Machine (LightGBM), and CatBoost Classifier.

Models were trained and tuned using the PyCaret ^20^ ML library (v3.1.0). We employed a stratified five-fold cross-validation scheme to report the average performance of models when trained on different subsets of the entire dataset.

To correct class imbalance in the training set, Synthetic Minority Over-Sampling Technique (SMOTE) ^21^ was utilized with validation test distribution of values kept as-is. Model hyperparameters were further tuned using grid search on each model type to yield a higher AUC that was calibrated to the outcome across propensity scores. The best model (highest AUC) was then set as the final model. The best model was found to be a LightGBM ^22^ model in all analyses. We reported ROC curves and AUC values for each fold and calculated the average AUC (± 1 standard deviation (SD)) where 1 = best classifier, 0.5 = random classifier. We also created a PR (precision-recall) curve for each time point with an AP (average precision) value (± 1 SD). Lastly, we reported “Variable Importance Scores” (relative rankings of weights placed on factors used to train the model) for the retrospective models, to help interpret what characteristics the models focused on at different prediction time points.

### 2.4 External Validation

Once models were trained and evaluated at UCLA, the associated code was sent to VUMC for local model training and testing. First, VUMC data sets were created and formatted to match the inputs of the UCLA model. Shared SQL queries ensured that the same data were extracted from the shared electronic health record vendor. Extracted clinical predictors were then manually mapped to ensure the raw data was of the expected values and format of the UCLA and inclusive of all similar VUMC data elements. There were several iterations of checking the dataset to verify VUMC applied the same patient inclusion criteria and procured the same set of clinical predictors. Once the data set was finalized, python scripts were executed locally to prepare the data for the model calibration and execution. All patient-level VUMC data remained on internal servers and only aggregated summary statistics were returned to the UCLA research team. Only the conservative set of inputs (as opposed to institutional set) was prepared for the VUMC model to simplify cross-center predictor variable mapping and limit predictions to those based only on highly standardized and widely available predictor variables. The three-group (trinary) prospective model was selected for external validation at VUMC as it represents the most difficult prediction scenario with three possible prediction outcomes as opposed to two.

## 3. Results

### 3.1 Demographics

Demographics and case counts are summarized in **Table I**. A total of 1,750 UCLA patients were included with an average age of 56 ± 20 years and 46% female. Verapamil was administered in 125 (7.1%) patients on average 7.6 ± 4.6 days after admission. A total of 1,654 VUMC patients were included with an average age of 53 ± 21 years, 42% female, 75 (4.5%) receiving verapamil. Patient age distributions at each institution were similar, with most patients being over 50 years of age (63% at UCLA, 59% at VUMC). Racial distributions differed most significantly in the portion of Caucasian individuals, being 41% at UCLA and 84% at VUMC. The distributions of body mass index (BMI) at each institution did not differ significantly.

### 3.2 Prospective Predictive Models

In Figure I, average ROC and PR curves for prospective UCLA models are reported. The binary prospective model achieved AUCs ranging from 0.68 (at t = 10 days of ICU data) to 0.88 (with t = 4 hours of ICU data) with the institutional model outperforming the conservative model by 0.00–0.03 AUC points on average. The trinary prospective model achieved AUCs in the range of 0.68–0.88 for predicting no CVRV, 0.73–0.83 for predicting CVRV < 3 days, and 0.50–0.88 for predicting CVRV in 3 + days across the 1-, 5-, and 10-day timepoints. In Figure II, LightGBM models are shown to outperform logistic regression at all timepoints. Additionally, theoretical CVRV rule-out performance is compared between models by examining precision when recall equals 1.00, with LightGBM outperforming logistic regression at all timepoints.

### 3.3 Retrospective Predictive Models

In Figure III, average ROC and PR curves for binary retrospective UCLA models are reported. Average AUCs ranged from 0.81–0.92 with the institutional models performing 0.01–0.09 AUC points better than the conservative models. In Figure IV, AUCs of the binary retrospective models are compared to those of a control retrospective logistic regression model; additionally, the predictor variables ranked by average importance score, and the temporal fluctuation of the top three predictor variables preceding vasospasm are shown.

### 3.4 External Validation

In Figure V, average ROC curves for the trinary prospective conservative VUMC models are reported. These models achieved AUCs ranging from 0.61–0.93 for predicting no CVRV, 0.81–0.82 for predicting CVRV < 3 days, and 0.48–0.88 for predicting CVRV in 3 + days across the 1-, 5-, and 10-day timepoints. Predictive models at VUMC performed very similarly to those at UCLA (on average 0.01 AUC points lower).

## 4. Discussion

We describe our experience using ML to predict CV requiring verapamil in subarachnoid hemorrhage patients using a diverse and temporally granular collection of ICU data at two separate institutions. Predictive models at both institutions achieved comparable and high predictive accuracy at multiple prediction time points, despite the patient populations differing appreciably in racial composition and being treated in separate health care systems with different decision pathways for verapamil administration.

### 4.1 Existing Literature

Literature characterizing the utility of artificial intelligence (AI) and ML for vasospasm prediction is limited. One systematic review from 2021 on AI use in neurocritical care ^23^ identified a single study using sparse dictionary learning and covariance-based features from digital subtraction angiography (DSA) to predict vasospasm with an AUC of 0.93, but this study was limited by a sample size of n = 22 and DSA availability ^17^. Another 2021 systematic review of ML for stroke diagnosis and outcome prediction ^24^ reported one study predicting DCI with an AUC of 0.74 across n = 317 patients ^12^, and one predicting a combination of DCI, angiographic vasospasm, or cerebral infarction with an accuracy of 95.2% but relied on radiography and matricellular protein lab availability ^13^.

Among other studies employing AI to predict CV, Dumont et al developed an artificial neural network (ANN) for predicting symptomatic cerebral vasospasm based on pre-existing clinical scores (Modified Fischer, Hunt and Hess) ^14^. The authors reported an AUC of 0.96, however this was only validated across n = 22 and relied on human-generated vasospasm risk scores. Skoch et al adapted Dumont’s ANN for pediatric populations and achieved similar performance over n = 16 patients ^15^. Roederer et al reported an AUC of 0.71 using a Naïve Bayes model on 81 acute SAH patients within two days of vasospasm ^16^. Kim et al predicted vasospasm defined as vessel stenosis > 50% with an AUC of 0.88 (n = 343) using a random forest ^10^ reliant on clinical grading scales and manually extracted image features. We found that average AUCs for cohorts under n = 1000 were associated with significant uncertainty, justifying the need for larger cohorts.

### 4.2 Prospective Predictive Models

Our prospective CVRV prediction model trained on raw ICU data alone achieved a high predictive accuracy (AUC = 0.88) over one week prior to verapamil injection and was validated over 1,750 patients, which is the largest ML-driven vasospasm prediction cohort by a factor of five in the known literature, and the largest vasospasm prediction cohort of any prediction methodology in the literature. Our conservative model achieved a similar AUC of 0.87. Perhaps most notably, the PR curves for prospective models were favorable with regards to rule-out of patients who will not need verapamil, ruling out CVRV in 8% of those not requiring verapamil up to 10 days in advance and without any false negatives. Typical ICU management involves serial neuro checks +/− transcranial dopplers (TCDs) with subsequent computed tomography angiography (CTA) if suspicion for vasospasm is high enough, followed by endovascular intervention if warranted ^5^. Knowing a patient are unlikely to progress to CVRV is invaluable for a few reasons: 1) frequency of neuro checks and TCD can be decreased in this low-risk population, and 2) patients in this category who exhibit neurologic deficit with or without elevated TCDs can be managed medically (BP augmentation) rather than skipping directly to CTA for fear of CVRV, therefore potentially cutting down on CTA frequency in this population.

The trinary prospective model achieved high predictive accuracy (average AUC = 0.86) when predicting the timeline of impending vasospasm. Such predictions may serve to alert providers when to initiate prophylactic BP augmentation, for example, therefore enabling preparatory clinical action in high risk individuals and titration of monitoring vigilance based on an individual’s CVRV risk. Because this is a prospective model like our previously described binary model, it can automatically generate new CVRV risk predictions every day for each patient in the ICU and guide care. Interestingly, as ICU admission progressed towards day 10, predictive accuracy declined slightly for the binary prospective model and more significantly for the trinary prospective model. We suspect these declines are attributable to the steadily decreasing portion of patients who are “obvious impending vasospasms” in the network’s eye.

By using only raw clinical data, our model inputs require zero human interpretation and can be fed clinical information without human supervision and update predictions in real-time. Prior attempts to predict vasospasm can be stratified into those which use only raw clinical data, and those which rely on clinical scores generated through human interpretation of diagnostic radiography. The former group has struggled to achieve predictive accuracy sufficient for clinical use ^7,25^. The other class of predictors ^9,10,13,26,27^ rely on human-generated clinical scores which are time-intensive and limited by subjectivity inherent to human interpretation of radiographic features. Such predictors are also not easily updated in real-time given the practical limitations on imaging frequency in the clinical setting. Additionally, our model is not proprietary and therefore readily implementable at any institution given all code is open source.

Our external validation supports the notion that our prospective models can be used in a multi-center context with nearly zero decline in peak predictive accuracy or CVRV rule-out performance. A logical next step will be to conduct a multi-center clinical study where it is prospectively predicted whether each patient will develop CVRV using our conservative model. Both the institutional and conservative models dramatically outperformed the control network, logistic regression, which assumes linearity between predictors and targets and is therefore performance limited in many settings. We therefore demonstrate a nonlinear relationship between the vast collection of ICU datapoints and the target.

### 4.3 Retrospective Predictive Models

Our study uniquely performed predictive modeling at multiple timepoints prior to the event. For the prospective model, doing so lends clinical utility in the form of daily risk CVRV predictions. With our retrospective model, performing predictions at multiple time points provides insight into the events leading to CVRV. Predictive accuracy began to decline appreciably in our retrospective model when predicting on a group of patients who were at least one week out from vasospasm. We believe the time interval prior to vasospasm at which retrospective predictive accuracy increases may represent the first detectable events in a pathophysiologic cascade towards vasospasm. To investigate this idea further, we analyzed the temporal fluctuation of predictor importance scores preceding verapamil in our retrospective conservative model.

Our analysis of the temporal fluctuation of vasospasm predictor importance scores may support previously hypothesized CV pathophysiology. After initial intracranial hemorrhage and ICP elevation, subarachnoid blood products are thought to trigger microglial activation and macrophage “crosstalk” leading to peripheral immune activation ^28^. Our model indicates that the predictive accuracy of maximum ICP peaks one week prior and then declines. This may explain the mixed results others have reported when attempting to predict CV with ICP alone within just a few days of onset ^16^. Our model shows that minimum white blood cell (WBC) count and maximum platelet count, markers of peripheral immune activation, rise in predictive accuracy approximately one week prior to vasospasm and immediately after maximum ICP importance score peaks. There is indeed considerable evidence that WBC and platelet counts are predictive of CV ^29,30^. The non-linear relationships between predictors and targets in ML do not allow us to infer that it is high, normal, low values, or some other characteristic entirely of any predictor which underlies its predictive accuracy. Therefore, the increases in the importance scores of minimum WBC and maximum platelet counts after maximum ICP predictive accuracy peaks may signal the initiation of the previously described peripheral immune activation following ICP elevation before CV.

### 4.4 External Validation

While predictive models have been widely published across fields of medicine, they are rarely implemented in routine clinical practice. Many reasons may underlie the failure to implement, though lack of external validation is a well-known contributing factor. We would largely agree that this is a critical step towards clinician acceptance. The process of externally validating our UCLA predictive models proved to be complex. Despite using a common electronic health record vendor, mapping clinical predictor variables from UCLA to VUMC was a lengthy process with much back-and-forth between respective teams, chiefly because each institution was blinded to all patient-level data of the other. It was necessary to have the VUMC clinical team verify clinical predictor variables were appropriately mapped, and a researcher with programming experience at VUMC to train and test the VUMC model. Given the anticipated complexity of the external validation process, we elected to test the most rigorous model configuration for simplicity, namely a model which prospectively predicted the previously described three-group outcome, using only the “conservative” set of clinical predictor variables. Our experience externally validating this model reinforces the notion that there is a large need in medicine to establish infrastructure to perform external validation quickly and easily.

### 4.5 Limitations

Inclusion criteria were based on ICD codes for SAH and ICU time, which are only available after discharge; in a subsequent prospective analysis, another surrogate for inclusion will be required. Because ML is a “black box” which draws non-linear relationships between predictors and targets, we cannot know which specific characteristic of the most predictive variables spawn their predictive accuracy. It should be noted that there was moderate class imbalance in the cohorts within this study, having more non-verapamil patients than verapamil patients. This is a difficult problem to avoid in this setting, where the true number of those receiving verapamil is low relative to those not receiving verapamil. As previously described, best efforts were made to account for said class imbalance using SMOTE and reporting average precisions. Finally, this study is subject to limitations characteristic of retrospective analyses and hence requires prospective validation.

## 5. Conclusion

Cerebral vasospasm is a prevalent and life-threatening complication of subarachnoid hemorrhage and requires vigilant clinical monitoring. Developing a reliable model for predicting CV has been an area of ongoing research interest and has proven to be challenging. We report a highly accurate ML-driven predictor of CV requiring verapamil in, to our knowledge, the largest and only multi-center study in the literature. Our ML model analyzes 172 unique raw ICU datapoints to accurately predict CV requiring verapamil on average a full week in advance. Further research will focus on prospective validation of this model and the prediction of lesser forms of vasospasm to further optimize hospital resource allocation in this setting.

## Figures and Tables

**Figure 1:**
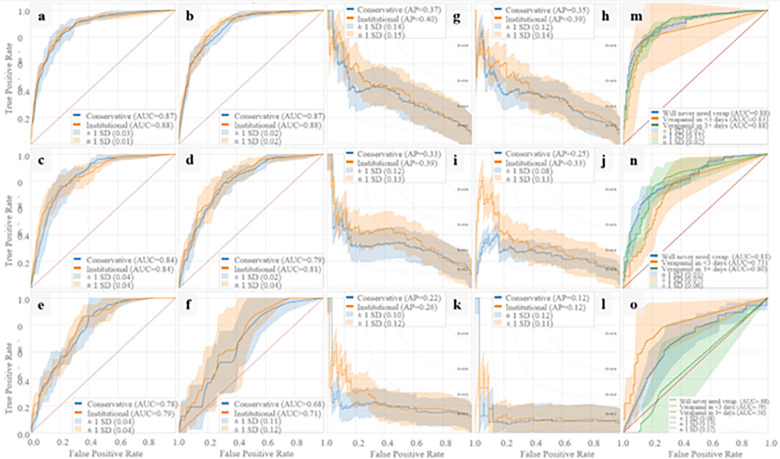
Prospective Model AUC and PR Curves Prospective model ROC and PR curves using five-fold cross validation. Binary prospective model results predicting CVRV or not during admission are shown in a-l. Panels a-f and g-l are AUCs and PR curves, respectively, using 4 hours, 1 day, 3 days, 5 days, 7 days, and 10 days of ICU data since ICU admission. Trinary prospective model results predicting CVRV in <=3 days, CVRV in >3 days, or no CVRV during admission, are shown in panels m-o, which show AUC curves using 1 day, 5 days, and 10 days of ICU data since ICU admission.

**Figure 2:**
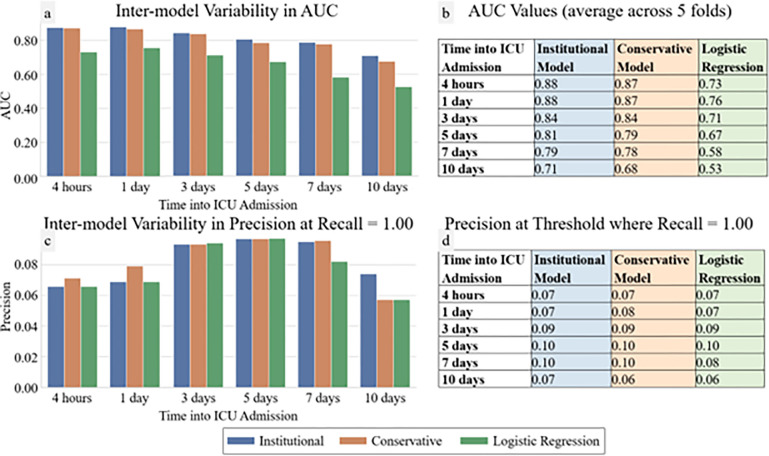
Prospective Model and Logistic Regression AUC and Theoretical CVRV Rule-Out Prospective institutional and conservative models and control model (logistic regression) AUCs and CVRV rule-out performance over time. Panels a and b display AUCs at each prediction timepoint for each model (a graphically, b in table format). Panels c and d show precisions at the threshold where Recall=1.00 (c graphically, d in table format).

**Figure 3:**
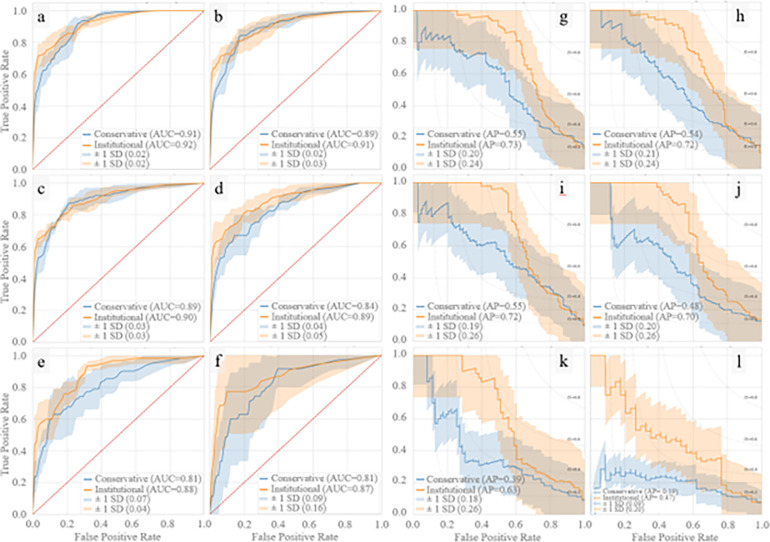
Retrospective Model AUC and PR Curves Retrospective model ROC and PR curves using five-fold cross validation. Binary retrospective model results predicting CVRV or not during admission are shown in panels a-l. Panels a-f and g-l are AUCs and PR curves, respectively, using 4 hours, 1 day, 3 days, 5 days, 7 days, and 10 days of ICU prior to the primary endpoint (CVRV or ICU downgrade).

**Figure 4:**
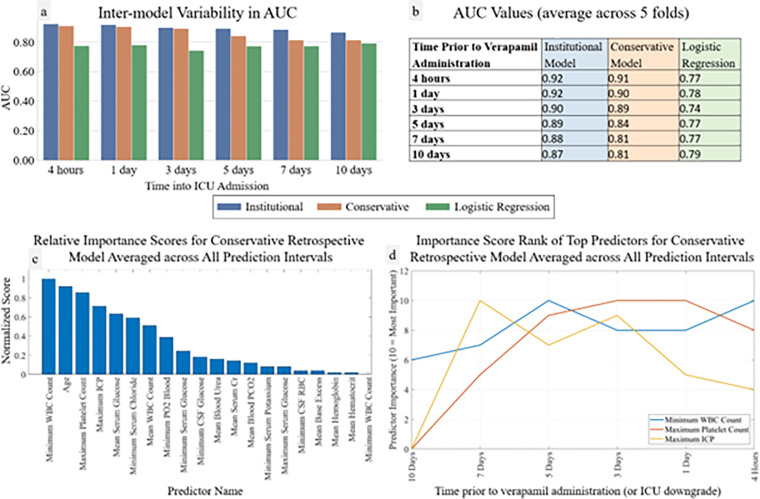
Retrospective Model and Logistic Regression AUCs and Importance Scores Retrospective institutional, conservative, and control (logistic regression) models AUCs over time alongside retrospective conservative model importance score analysis. Panels a and b display AUCs at each prediction timepoint for each model (a graphically, b in table format). Panels c and d show importance score analysis: c shows averaged ranked variable importance scores over all prediction timepoints and d shows temporal fluctuation in importance of the three predictor variables with the overall highest variable importance scores (10 = #1 overall predictor, 9 = #2 overall predictor, etc).

**Figure 5:**
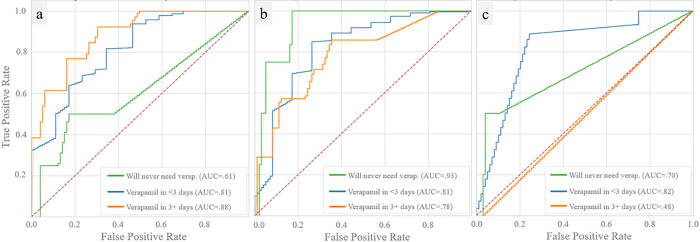
External Validation of Vasospasm Prediction Prospective, trinary model trained and tested on the conservative set of clinical predictor variables from the VUMC dataset. Panel a displays ROCs and AUCs of five-fold cross validations for each group with the model trained on 1 day of ICU data, panel b with 5 days of ICU data, and panel c with 10 days of ICU data.

## References

[1] Dorsch N. W. C. & King M. T. A review of cerebral vasospasm in aneurysmal subarachnoid haemorrhage Part I: Incidence and effects. J Clin Neurosci 1, 19–26 (1994). 18638721 10.1016/0967-5868(94)90005-1

[2] EEG Monitoring to Detect Vasospasm after Subarachnoid Hemorrhage |…https://www.reliasmedia.com/articles/34004-eeg-monitoring-to-detect-vasospasm-after-subarachnoid-hemorrhage.

[3] Frontera J. A. Defining Vasospasm After Subarachnoid Hemorrhage. Stroke 40, 1963–1968 (2009). 19359629 10.1161/STROKEAHA.108.544700

[4] Dabus G. & Nogueira R. G. Current Options for the Management of Aneurysmal Subarachnoid Hemorrhage-Induced Cerebral Vasospasm: A Comprehensive Review of the Literature. Interv Neurol 2, 30 (2013). 25187783 10.1159/000354755 PMC4031782

[5] Diringer M. N. Critical care management of patients following aneurysmal subarachnoid hemorrhage: recommendations from the Neurocritical Care Society’s Multidisciplinary Consensus Conference. Neurocrit Care 15, 211–240 (2011). 21773873 10.1007/s12028-011-9605-9

[6] Otite F. Impaired cerebral autoregulation is associated with vasospasm and delayed cerebral ischemia in subarachnoid hemorrhage. Stroke 45, 677–682 (2014). 24425120 10.1161/STROKEAHA.113.002630 PMC4415505

[7] Przybycien-Szymanska M. M. & Ashley W. W. Biomarker Discovery in Cerebral Vasospasm after Aneurysmal Subarachnoid Hemorrhage. J Stroke Cerebrovasc Dis 24, 1453–1464 (2015). 25957908 10.1016/j.jstrokecerebrovasdis.2015.03.047

[8] Ishihara H. Hounsfield Unit Value of Interpeduncular Cistern Hematomas Can Predict Symptomatic Vasospasm. Stroke 51, 143–148 (2020). 31694506 10.1161/STROKEAHA.119.026962

[9] Frontera J. A. Prediction of symptomatic vasospasm after subarachnoid hemorrhage: the modified fisher scale. Neurosurgery 59, 21–26 (2006). 16823296 10.1227/01.neu.0000243277.86222.6c

[10] Kim K. H., Koo H. W., Lee B. J. & Sohn M. J. Analysis of risk factors correlated with angiographic vasospasm in patients with aneurysmal subarachnoid hemorrhage using explainable predictive modeling. J Clin Neurosci 91, 334–342 (2021). 34373049 10.1016/j.jocn.2021.07.028

[11] Li J., Zhou K., Wang L. & Cao Q. Predictive Model of Cerebral Vasospasm in Subarachnoid Hemorrhage Based on Regression Equation. Scanning 2022, (2022). 10.1155/2022/3397967 PMC9064499 35581969

[12] Ramos L. A. Machine learning improves prediction of delayed cerebral ischemia in patients with subarachnoid hemorrhage. J Neurointerv Surg 11, 497–502 (2019). 30415227 10.1136/neurintsurg-2018-014258

[13] Tanioka S. Machine Learning Analysis of Matricellular Proteins and Clinical Variables for Early Prediction of Delayed Cerebral Ischemia After Aneurysmal Subarachnoid Hemorrhage. Mol Neurobiol 56, 7128–7135 (2019). 30989629 10.1007/s12035-019-1601-7

[14] Dumont T. M., Rughani A. I. & Tranmer B. I. Prediction of symptomatic cerebral vasospasm after aneurysmal subarachnoid hemorrhage with an artificial neural network: feasibility and comparison with logistic regression models. World Neurosurg 75, 57–63 (2011). 21492664 10.1016/j.wneu.2010.07.007

[15] Skoch J. Predicting symptomatic cerebral vasospasm after aneurysmal subarachnoid hemorrhage with an artificial neural network in a pediatric population. Childs Nerv Syst 33, 2153–2157 (2017). 28852853 10.1007/s00381-017-3573-0

[16] Roederer A., Holmes J. H., Smith M. J., Lee I. & Park S. Prediction of significant vasospasm in aneurysmal subarachnoid hemorrhage using automated data. Neurocrit Care 21, 444–450 (2014). 24715326 10.1007/s12028-014-9976-9

[17] Capoglu S., Savarraj J. P., Sheth S. A., Choi H. A. & Giancardo L. Representation Learning of 3D Brain Angiograms, an Application for Cerebral Vasospasm Prediction. Annu Int Conf IEEE Eng Med Biol Soc 2019, 3394–3398 (2019). 31946608 10.1109/EMBC.2019.8857815

[18] Kim K. H., Koo H. W., Lee B. J. & Sohn M. J. Analysis of risk factors correlated with angiographic vasospasm in patients with aneurysmal subarachnoid hemorrhage using explainable predictive modeling. J Clin Neurosci 91, 334–342 (2021). 34373049 10.1016/j.jocn.2021.07.028

[19] Epstein R. H., Hofer I. S., Salari V. & Gabel E. Successful Implementation of a Perioperative Data Warehouse Using Another Hospital’s Published Specification From Epic’s Electronic Health Record System. Anesth Analg 132, 465–474 (2021). 32332291 10.1213/ANE.0000000000004806

[20] Home - PyCaret. https://pycaret.org/.

[21] Chawla N. v., Bowyer K. W., Hall L. O. & Kegelmeyer W. P. SMOTE: Synthetic Minority Over-sampling Technique. Journal of Artificial Intelligence Research 16, 321–357 (2002).

[22] Ke G. LightGBM: A Highly Efficient Gradient Boosting Decision Tree.

[23] Azimi P. Artificial neural networks in neurosurgery. J Neurol Neurosurg Psychiatry 86, 251–256 (2015). 24987050 10.1136/jnnp-2014-307807

[24] Mainali S., Darsie M. E. & Smetana K. S. Machine Learning in Action: Stroke Diagnosis and Outcome Prediction. Front Neurol 12, 2153 (2021). 10.3389/fneur.2021.734345 PMC8685212 34938254

[25] Zhang Y., Clark J. F., Pyne-Geithman G. & Caruso J. Metallomics study in CSF for putative biomarkers to predict cerebral vasospasm. Metallomics 2, 628–637 (2010). 21072354 10.1039/c0mt00005a

[26] Friedman J. A. Volumetric quantification of Fisher Grade 3 aneurysmal subarachnoid hemorrhage: a novel method to predict symptomatic vasospasm on admission computerized tomography scans. J Neurosurg 97, 401–407 (2002). 12186469 10.3171/jns.2002.97.2.0401

[27] Hickmann A. K. The value of perfusion computed tomography in predicting clinically relevant vasospasm in patients with aneurysmal subarachnoid hemorrhage. Neurosurg Rev 36, 267–278 (2013). 23104502 10.1007/s10143-012-0430-1

[28] Small C. Microglia and Post-Subarachnoid Hemorrhage Vasospasm: Review of Emerging Mechanisms and Treatment Modalities. Clinical surgery journal 3, (2022).

[29] McGirt M. J. Leukocytosis as an independent risk factor for cerebral vasospasm following aneurysmal subarachnoid hemorrhage. J Neurosurg 98, 1222–1226 (2003). 12816268 10.3171/jns.2003.98.6.1222

[30] Aggarwal A. Vasospasm following aneurysmal subarachnoid hemorrhage: Thrombocytopenia a marker. J Neurosci Rural Pract 4, 257–261 (2013). 24250155 10.4103/0976-3147.118762 PMC3821408

